# How to Face the Advent of SARS-CoV-2 Vaccination in IBD Patients: Another Task for Gastroenterologists

**DOI:** 10.3390/vaccines9030248

**Published:** 2021-03-12

**Authors:** Alfredo Papa, Franco Scaldaferri, Lorenzo Maria Vetrone, Matteo Neri, Antonio Gasbarrini, Loris Riccardo Lopetuso

**Affiliations:** 1 CEMAD Digestive Disease Center, Fondazione Policlinico Universitario A. Gemelli IRCCS, 00168 Roma, Italy; francoscaldaferri@gmail.com (F.S.); vetrone.md@gmail.com (L.M.V.); antonio.gasbarrini@unicatt.it (A.G.); lopetusoloris@libero.it (L.R.L.); 2 Dipartimento di Scienze Mediche e Chirurgiche, Università Cattolica del Sacro Cuore, 00168 Roma, Italy; 3 Department of Medicine and Ageing Sciences, “G. d’Annunzio” University of Chieti-Pescara, 66100 Chieti, Italy; mneri@unich.it; 4 Center for Advanced Studies and Technology (CAST), “G. d’Annunzio” University of Chieti-Pescara, 66100 Chieti, Italy

**Keywords:** SARS-CoV-2, vaccines, IBD, COVID-19, acceptance

## Abstract

The severity of the COVID-19 pandemic has led to an unprecedented effort to develop vaccines against SARS-CoV-2 infection since this seems to be the most effective strategy to counter the pandemic. In the past weeks, the administration of vaccines has started in different parts of the world sustaining the hype of significantly containing the impact of SARS-CoV-2 infection. However, the rapid time lapse from vaccine development to distribution has raised several concerns on its safety and efficacy. This topic is particularly felt by patients with chronic conditions and immumodulating therapies that could compromise their immune system such as inflammatory bowel disease (IBD). Here, we explore the potential future implications of the SARS-CoV-2 vaccines introduction in the IBD field, touching upon the clinical experience coming from available data on vaccinations against other infections. We also dissect the factors associated with the acceptability of SARS-CoV-2 vaccination, describing the possible strategies that gastroenterologist should adopt to reach the highest rate of vaccinations in IBD patients.

## 1. Introduction

The COVID-19 pandemic has caused more than 108 million cases and 2 million deaths worldwide [1]. The severity of the situation has led to an unprecedented effort to develop and test dozens of vaccines against SARS-CoV-2 infection. This seems to be the most effective and quick strategy to counter the pandemic. Currently, 18 SARS-CoV-2 vaccines have reached the final stages (e.g., phase III clinical trials) and of these, three have been already approved for full use or emergency/early use after substantial data of efficacy and safety [2,3,4]. Other vaccines have been authorized in Russia and China, but without waiting for the results of Phase III trials, thus raising doubts on their reliability. In the last weeks, the administration of the specific vaccines has started in the United Kingdom, Europe, United States, Canada, and other Countries, initially favoring healthcare personnel, care-home staff and residents together with elderly subjects. This certainly feeds the hype of significantly containing the impact of SARS-CoV-2 infection However, the extremely fast time lapse from vaccine development to distribution is unprecedented and this has raised several questions not only from the medical staff but also from the general public opinion [5]. This topic is particularly felt by patients with underlying conditions that compromise their immune system such as inflammatory bowel disease (IBD), including Crohn’s disease (CD), and ulcerative colitis (UC) patients.

Here, we explore the potential future implications of the SARS-CoV-2 vaccines introduction in the IBD field, touching upon the clinical experience coming from available data on vaccinations against other viral infections. We will also dissect the factors that will be eventually associated with the acceptability of SARS-CoV-2 vaccination in IBD patients.

## 2. IBD and Vaccines

During the course of disease, the majority of IBD patients need long-term immuno-modulatory therapy and thus are at increased risk for infectious diseases. Many of these diseases are easily preventable by vaccination. A detailed summary of the main vaccines recommended in IBD patients according to international guidelines is reported in Table 1 [6,7,8,9]. In IBD patients not treated with immunosuppressants, there are no particular contraindications for the use of inactivated or live vaccines. Conversely, in immunosuppressed patients the differentiation between live vaccines and inactivated vaccines is crucial. For live vaccines (measles, mumps, and rubella, varicella, Herpes Zoster, yellow fever) the class of immunosuppressive drug, their dosage, and the timing after their suspension can play a role in the administration strategies [9]. On the other side, inactivated vaccines can be managed differently. They can be easily administered independent of immunosuppressive therapy without concerns about medication group or dosage. However, they may be less immunogenic and therefore vaccine-specific administration strategies are recommended (Table 1).

Indeed, to maximize their efficacy, vaccination should be preferentially dispensed during disease remission, at least 4 weeks before starting the immunosuppressive therapy or at its lowest possible dose. Moreover, the immune response to a booster vaccine administered during immunosuppressive therapy is less disturbed than that to a primary vaccine administration. Conjugate vaccines may be favorably given rather than polysaccharide vaccines since they are able to promote higher affinity antibody production with longer lasting immune and memory responses. Finally, when available, immunosuppressed patients may be usefully verify their immunization through specific serology tests after the completion of a primary vaccination [9]. We believe that these strategies can be considered and applied also for the upcoming SARS-CoV-2 vaccines.

In this scenario, the vaccination status and dedicated serological tests should be assessed as soon as the disease is diagnosed and missing vaccinations should be prescribed, preferably before the immunosuppressive regimen is started. Despite these recommendations, several studies have reported low administration rates of advised vaccinations in IBD cohorts [9,10,11]. Main reasons for non-immunization comprised lacking knowledge and concerns about potential side effects both among patients and physicians. Another problem could be connected to a potential disagreement on the responsibility for who is in charge for vaccines prescription (e.g., primary care physicians or gastroenterologists). Overall, gastroenterologists should be responsible for registering the vaccination history and completing the immunizations when needed. Moreover, the majority of IBD patients is of young age and might attend the gastroenterologist more frequently than primary care physicians with more possibilities of updating the vaccination status. The gastroenterologist would also be responsible for specific recommendations on traveling vaccinations. Furthermore, household members and close contacts should be also considered and vaccinated against highly contagious infectious disease to prevent disease transmission to IBD subjects.

## 3. COVID-19 and IBD

So far, several reports have shown that the incidence of COVID-19 in IBD patients is not different from that found in the general population [12]. Furthermore, the clinical course of COVID-19 appeared no more severe in IBD patients, even in those treated with a biological therapy, compared with controls without IBD [13]. Interestingly, the risk factors associated to a worse prognosis of COVID-19 in IBD patients are the same as the general population (i.e., advanced age and the presence of comorbidities) with the addition of active disease at the time of infection [14]. However, these reassuring findings are largely due to the health agencies recommendations for reducing COVID-19 spread, to the IBD treatments adequately prescribed by gastroenterologists as well as to the cautious preventive measures adopted by the patients [15]. In fact, especially during the first months of the pandemic, access to diagnostic exams (i.e., endoscopy), hospital admissions or outpatient visits with the sole exception of infusion therapy and emergencies have been deferred or canceled [16,17]. However, with the reorganization of hospital, work, school and social activities, despite the implementation of specific limitations, the spread of SARS-CoV-2 infection in IBD patients and in the general population has increased again in the absence of therapies and preventive measures. In addition, the partial or total closures of the healthcare services lead to detrimental consequences for IBD subjects as well as for patients with others chronic or neoplastic diseases, both in terms of diagnostic delay and prognosis [18].

Therefore, the advent of SARS-CoV-2 vaccinations should represent an opportunity to catch with enthusiasm and confidence with the end goal of quickly promoting health and safety, and restoring the best possible treatments for IBD patients.

## 4. The Arrival of SARS-CoV-2 Vaccines

More than 100 candidate SARS-CoV-2 vaccines are actually under development. Most of them are targeting the surface membrane S-protein that is involved in receptor binding, membrane fusion and entry into host cells [19]. Among the different vaccine platforms used so far, the mRNA-based SARS-CoV-2 vaccines have been the most attractive and the first ones to be approved by the American and European regulatory agencies (i.e., Food and Drug Administration (FDA) and European Medicines Agency (EMA)), probably owing to their rapid and low-cost manufacturing process. mRNA-based vaccines have generally favorable safety profiles, without risk of insertional mutagenesis since they do not require the cellular nucleus to express the antigen. Moreover, there is no risk of infection spread because there is no need to handle the virus during manufacturing. They have been already used to obtain vaccines against other infectious diseases, such as Zika virus, influenza virus, respiratory syncytial virus (RSV), Ebola virus, and HIV [19,20,21,22]. On the other side, their intrinsic mRNA structure requires special attention for guaranteeing strict storage settings in order to maintain stability and translational efficiency [19]. In this context, two mRNA-based vaccines have been already licensed for commercial use after the evaluation of their respective phase III clinical trials. mRNA-1273 and BNT162b2 are lipid nanoparticle-formulated nucleoside-modified RNA vaccines that encode a prefusion stabilized membrane-anchored SARS-CoV-2 full-length spike protein. They should be able to induce both humoral and cellular immunity in the base of animal studies and if phase I clinical trials [19,23,24,25,26]. The mRNA-1273 vaccine sustained a 94.1% efficacy, while a two-dose regimen of BNT162b2 showed 95% protection at preventing COVID-19 illness, including severe disease [2]. Aside from transient local and systemic reactions, no safety concerns were identified [2].

Beside these, viral vector-based SARS-CoV-2 vaccines have been also developed. They are able to promote robust immune responses and can induce both humoral and cellular immunity [27]. In particular, adenovirus vectors can be easily grown to high titers in cell lines, possess elevated transduction efficiency and transgene expression, and show a broad range of viral tropism [19]. At an interim analysis of phase III trial, S protein-expressing chimpanzee adenovirus-based vaccine (ChAdOx1 nCoV-19) exhibited an acceptable safety profile and an overall efficacy of 70.4% [4]. This vaccine has been already commercialized in Great Britain, while is waiting for the final approval by FDA and EMA.

In this scenario, the intrinsic immune-mediated nature and the need for immunomodulating or biological therapies in IBD patients have led to several questions on the safety and the efficacy of the SARS-CoV-2 vaccines [28]. First, we believe, that given the unprecedented development rapidity of these vaccines, the answers on their performance in particular groups of patients (e.g., IBD patients) will be only addressed over time. On the one hand, the efficacy data coming from phase III trials certainly have sustained the hype of significantly containing the impact of SARS-CoV-2 infection. Conversely, no specific data for IBD patients are yet available in these reports. So we still need to extrapolate concepts of safety and efficacy from studies conducted in IBD populations with different vaccines. Undoubtedly, new accumulating reports obtained from real-world data of vaccinated patients in appropriate post-marketing registers with eventual adverse reactions will clarify their safety both in the short and especially in the long term. Moreover, the possibility of having different vaccines obtained from distinctive platforms will also allow their comparison and therefore the identification of the most effective and safe solution according to demographic characteristics and to comorbidities, including IBD. Certainly, the main risk in IBD patients treated with biologic agents or small molecules is the potential concern of suboptimal vaccine responses rather than vaccine adverse effects. Nevertheless, the risk of morbidity and mortality linked to the COVID-19 complications far outweighs the apprehension of data uncertainty in a small cohort of subjects. In accordance with previous vaccine experiences, it is reasonable to accept that any vaccination should be completed prior to initiating immunosuppressive drugs [29]. In patients treated with immunosuppressant, SARS-CoV-2 vaccination should be also recommended based on a favorable risk/benefit ratio and sustained by an apparently encouraging safety profile and a clinically significant risk of hospitalization, complications, and death associated with COVID-19 [29]. In this setting, the monitoring of the antibody titer after vaccination could be useful. These considerations strongly sustain SARS-CoV-2 vaccination in patients with IBD.

## 5. Factors Associated with Increased Likelihood of Accepting COVID-19 Vaccination

As stated above, more than one SARS-CoV-2 vaccine will be available from the end of 2020 and the beginning of 2021. Therefore, the primary goal of the World Health Organization (WHO) and local health systems will be to vaccinate the largest proportion of the population to achieve herd immunity and ultimately prevent wider SARS-CoV-2 spread [30]. However, especially in the coming months, the limited number of available vaccine doses and the organization of logistics will require a priority list for carrying out the vaccinations. Accordingly, the vaccination campaign has started at the beginning of December 2020 from the front-line health-care personnel, care-home staff and residents [31] and will consequently involve the elderly and patients with chronic conditions including IBD.

Consequently, a difficult future task to face for gastroenterologists will be to get the highest acceptance rate of SARS-CoV-2 vaccination in their IBD patients. Recently, factors associated with the likelihood of accepting SARS-CoV-2 vaccination have been extensively studied in large population cohorts in order to drive public health information campaigns and to address vaccine hesitancy [32,33]. In these surveys, multiple demographic, socio-economic and health-related variables have been included, as well as vaccine characteristics such as different hypothetical thresholds of efficacy or side effects or even the country of production (e.g., lower acceptance rate for vaccines made in China compared to vaccines produced in the United States) [32,33]. Some of these variables are unchangeable, but there are others that, if implemented, would significantly reduce SARS-CoV-2 vaccine hesitancy. In particular, the endorsements of recognized health organizations (i.e., Centers for Disease control and prevention or WHO) or renowned national universities and medical institutions with their distinguished specialists could be part of public outreach campaigns in order to overcome the doubts of reluctant patients. Indeed, the involvement of the health care staff in recommending SARS-CoV-2 vaccine appears among the most significant aspects in driving the compliance to vaccination [34]. This means that an essential task in reducing IBD patients’ hesitancy will be played by gastroenterologists who will have to educate and inform their patients on the favorable risk-benefit ratio of SARS-CoV-2 vaccine, as already reported for other vaccine recommendations [35]. On this point, we think that an overt statement by health care professionals (e.g., through the use of “I’m vaccinated” pins or stickers or social media campaigns) would be a crucial encouraging tool to guide the compliance of patients [36]. In fact, making a commitment to take a recommended action (e.g., getting vaccinated) is much more effective at convincing others than simply recommending that specific action [36].

However, as part of an exhaustive information, gastroenterologists will have to alert patients that the vaccine does not give 100% protection against COVID-19 and that, at the moment, we are not sure that it prevents the transmission of SARS-CoV-2 infection [37]. Therefore, it will be essential to follow the preventive measures taken so far until herd immunity will be achieved. Finally, we want to underline the role of patients’ associations in providing correct information to IBD patients on vaccine safety and efficacy, especially in the era of fake news and disinformation. A comprehensive list of the actions and strategies that could be useful to increase the acceptance of COVID-19 vaccine is summarized in Table 2.

## 6. Conclusions

SARS-CoV-2 vaccination represents a key milestone on the steep path toward the ultimate challenge: defeating the SARS-CoV-2 pandemic and come back to a “normal” life. To this end, it is essential that the majority of the population will get vaccinated. IBD patients, as those affected by other immune-mediated inflammatory diseases, will have an early access to the vaccine. Consequently, gastroenterologists must make every effort for obtaining the highest consent to SARS-CoV-2 vaccination. A detailed description of its favorable risk-benefit ratio based on transparent efficacy data together with the reassurance of vaccine safety will be a crucial task to be implemented. Obviously, a vigilant surveillance on vaccine efficacy and safety, especially in the long term, is necessary, taking also into account that data in subgroups of patients with chronic diseases or during immunosuppressant therapies are still largely lacking [38]. Finally, we strongly believe that the endorsement of healthcare professionals vaccination along with the national and global healthcare organizations are essential to ensure satisfactory vaccine acceptance.

## Figures and Tables

**Table 1:** Main vaccines recommended for patients with inflammatory bowel disease (IBD).

Infectious Agent	Type of Vaccine	Target Population	Dosage	Efficacy and/or Safety Concerns
Influenza	Inactivated vaccine	All patients	Annual vaccine with trivalent	Close contacts should be vaccinated; possible reduced immunogenicity during IT
Hepatitis B	Inactivated vaccine	All seronegative patients	3 doses at 1,1–2 and 4–6 months	Check antibodies titers 1 month after the last dose; if no response revaccinate or double dose HBV vaccine
HPV	Inactivated vaccine	Female and male; 11 to 26 years of age	3 doses: 0, 2, and 6 months	Use HPV4 vaccine (quadrivalent)
S. pneumoniae	Inactivated vaccine	All patients	PCV13 followed by a dose of PPSV23 after 8 weeks; re-vaccinate with a single dose of PPSV23 5 year after	Another dose of PPSV23 should be administered at age 65 years or older (if at least 5 years have elapsed since the previous PPSV23 dose)
Varicella	Live attenuated vaccine	All seronegative patients	2 doses (>4 weeks apart) at least 3 weeks before starting IT	Contraindicated during IT
Herpes zoster	Inactivated vaccine	All patients	Two doses: 0 and 2–6 months	Safe and immunogenic in immunosuppressed patients

Abbreviations: IBD, inflammatory bowel disease; IT, immunosuppressive therapy; HBV, hepatitis B virus; HPV, human papillomavirus; HPV4, quadrivalent HPV vaccine; PCV, pneumococcal conjugate vaccine; PPSV, pneumococcal polysaccharide vaccine; S. pneumoniae: Streptococcus pneumoniae.

**Table 2:** Possible strategies for increasing adherence to SARS-CoV-2 vaccination in IBD patients.

Strategy	Type of Intervention
Information and education	Gastroenterologists and nurses should inform their IBD patients about the favorable risk-benefit ratio of accepting COVID-19 vaccination, also by means of educational materials including posters or booklets available in outpatients’ clinics
Vaccination endorsement by global and national health organizations	WHO, CDC, and national health institutions should publish on their website recommendation to get vaccinated for individuals belonging to “at risk” categories (such as patients with immune-mediated inflammatory diseases, including IBD)
Informative campaigns and vaccination recommendations by national and international medical societies with interest in IBD	ECCO, IOIBD, CCF, and other national societies/groups for the study of IBD should publish on their website and/or on gastroenterology or internal medicine journals the recommendations or guidelines for COVID-19 vaccine promotion
Social media informative campaigns	Renowned medical institutions (hospitals and universities) and distinguished physicians could be part of the public outreach campaigns for COVID-19 vaccine promotion by means of institutional or personal social media (e.g., Twitter, Linkedin, Facebook)
Overt statement by healthcare professionals of having been COVID-19 vaccinated	Reporting the own vaccination status (e.g., by “I’m vaccinated” pins or stickers or social media campaigns) would be an important motivating tool to guide the patient’s choice of vaccination
Patients’ associations endorsement	Direct patient involvement is a crucial educational strategy, which is mainly accomplished through patient associations (and their websites) as well physician counseling
Outcome and safety registries to monitor real-life performance of COVID-19 vaccines	To collect and supply real-life COVID-19 vaccine efficacy and safety data, with special emphasis to IBD patients

Abbreviations: COVID-19, Coronavirus disease-2019; IBD, inflammatory bowel disease; WHO, World Health Organization; CDC, Centers for Disease Control and Prevention; ECCO, European Crohn’s and Colitis Organization; IOIBD, International Organization for the study of Inflammatory bowel disease; CCF, Crohn’s and Colitis Foundation.

## Data Availability

No new data were created or analyzed in this study. Data sharing is not applicable to this article.
